# 

**Published:** 1892-03

**Authors:** 


### HALL'S JOURNAL of HEALTH

ESTABLISHED 1854

CONTENTS

Vol. 39. No. 3.

| | PAGE. |
|---|---|
| La Grippe | 49 |
| A Queer Matrimonial Mart | 52 |
| Talks with Dr. Mandeville | 54 |
| Animals which never see Daylight | 55 |
| Heredity in Monkeys | 57 |
| What Salt Will Do | 58 |
| Treatment of a Cold | 58 |
| Prevention of Influenza | 59 |
| Women and their Duty | 60 |
| Thought and the Brain | 61 |
| A Swim in Salt Lake | 64 |
| What Ants Can Do | 65 |
| Peculiarities of the Pulse | 67 |
| Miscellaneous | 68 |
| Literary | 71 |
| Sun and Shade | 72 |

INDEX TO ADVERTISEMENTS OF HALF A PAGE AND OVER.

| | PAGE. |
|---|---|
| Cornish & Co | II |
| Bovinine | IV |
| Dr. J. C. Ayer & Co | V |
| Hall's Journal of Health | VI |
| Hall's Journal Premiums | VII |
| Magee Emulsion Co | X |
| Herba Vita | X |
| Royal Baking Powder | XI |
| Buffalo Lithia Water | XI |

